# White Matter Microstructural Alterations over the Year after Acute Ischemic Stroke in Patients with Baseline Impaired Cognitive Functions

**DOI:** 10.1155/2023/6762225

**Published:** 2023-07-07

**Authors:** Bingyuan Wu, Shida Guo, Xiuqin Jia, Zuojun Geng, Qi Yang

**Affiliations:** ^1^Department of Radiology, Beijing Chao-Yang Hospital, Capital Medical University, Beijing 100020, China; ^2^Department of Radiology, The Second Hospital of Hebei Medical University, Shijiazhuang 050000, Hebei, China

## Abstract

**Background:**

The disruption of white matter (WM) integrity is related to poststroke cognitive impairment (PSCI). The exploration of WM integrity alterations in the chronic stage of acute ischemic stroke (AIS) may help to improve the long-term outcomes of PSCI.

**Methods:**

Sixty patients showing impaired cognitive functions within 3 days after AIS (baseline) and 25 healthy controls underwent diffusion kurtosis imaging scan and cognitive assessment at baseline and 1 year. Based on the tract-based spatial statistics (TBSS), kurtosis fractional anisotropy (KFA) and mean kurtosis (MK) were compared in WM tracts between the groups.

**Results:**

One year after AIS, 25 patients were diagnosed with PSCI and 35 patients with non-cognitive impairment (NCI). Compared with baseline, cognitive performance improved in 54 patients and remained unchanged in 6 patients at 1 year. TBSS analysis showed that there were no significant differences in WM tract integrity between the AIS and control groups at baseline (*P* > 0.05). Compared with the control group, the KFA and MK in multiple WM tracts in the AIS group decreased significantly at 1 year (*P* < 0.05). Longitudinal analysis showed that the KFA and MK of multiple WM tracts recorded at 1 year were significantly lower than those recorded at baseline in the AIS, PSCI, and NCI groups (*P* < 0.05), and PSCI group had a faster degeneration than NCI group (*P* < 0.05).

**Conclusion:**

The finding suggests that the patients with baseline impaired cognitive functions still have WM microstructural damages at 1 year poststroke, even if their cognitive function has improved or returned to normal. Cautions should be taken against the possible negative impact of these changes on long-term cognition.

## 1. Introduction

With the decrease of the mortality rate of acute ischemic stroke (AIS), poststroke cognitive impairment (PSCI) is attracting more and more attention. Contrary to the linear decrease seen in cognitive changes in neurodegenerative diseases, stroke survivors appear to follow different trajectories, with improved, deteriorated, or unchanged over time [1, 2]. There are many factors associated with the occurrence and evolution of PSCI, including age, prestroke cognitive status, brain resilience, comorbid neurodegenerative, and cerebral small vessel disease burden [2, 3]. Therefore, reducing the interference factors in the study is conducive to a better exploration of the pathological mechanism of PSCI and facilitates personalized treatment and optimal prevention.

White matter (WM) microstructural damage is an important neuroradiological predictor of PSCI [4–6]. At present, there are many studies [5, 7, 8] on the damages of WM integrity in subacute stage and its correlations with PSCI. However, the WM integrity profiles have been sparsely investigated in poststroke recovery studies, and prolonged follow-up is needed to investigate WM integrity and its impact on long-term cognitive outcomes. Diffusion kurtosis imaging (DKI), with the diffusion being non-Gaussian, can better reveal subtle impairments in normal-appearing WM microstructure prior to conventional magnetic resonance imaging (MRI) detection [5, 9–11]. The decrease of kurtosis fractional anisotropy (KFA) implicates demyelination or axonal disruption of nerve fibers, and reduced mean kurtosis (MK) in WM is likely related to impoverished cell compartmentalization and an increase in membrane permeability [5]. In this study, we selected relatively young patients showing impaired cognitive functions within 3 days after AIS onset and minimized the interference of neurodegenerative factors, aimed at evaluating the evolution of WM tract integrity over the year following AIS using tract-based spatial statistics (TBSS) analysis based on DKI.

## 2. Materials and Methods

### 2.1. Study Design and Participants

A consecutive study was approved by the Medical Research Ethics Committee and conducted at the corresponding hospital between 2018 and 2020. Written informed consent was acquired from all participants. The study consisted of 60 enrolled patients (42 males and 18 females, 57.70 ± 7.33 years) with acute ischemic stroke as the AIS group. The inclusion criteria included: (1) diagnosis of the first AIS, clinical assessment, and MRI within 3 days after stroke onset; (2) National Institutes of Health Stroke Scale (NIHSS) score at admission between 1 and 15; (3) primary school and higher education; (4) no prestroke cognitive decline; (5) baseline impaired cognitive functions at the admission assessment. The exclusion criteria included: (1) previous history of cognitive impairment (CI), central nervous system disease, psychosis, and other related medical conditions; (2) other factors that interfere with clinical and imaging evaluations; (3) MRI medial temporal lobe atrophy (MTA) score ≥1.5 at admission. Twenty-five healthy controls (HC) (16 males and 9 females, 56.16 ± 6.96 years) matched with patients according to age, gender, and education were recruited, without a history of stroke, mental disorder, and CI. All participants were right-hand dominance.

### 2.2. Clinical Assessment

All participants in the AIS and HC groups underwent two neuropsychological assessments at baseline and 1 year by two experienced neurologists during a medical visit, including the mini-mental state examination (MMSE) and Montreal Cognitive Assessment (MoCA). One point was added to MoCA score if the subject had ≤12 years of education. Prestroke cognitive status was evaluated with the Informant Questionnaire in Cognitive Decline in the Elderly (IQCODE).

The global cognitive function was assessed using MMSE and MoCA scores. MMSE score ≥27 and MoCA score ≥26 indicated normal cognitive function. MoCA score <26 indicated CI. The CI severity was rated according to the MMSE score: mild (≥21), moderate (10–20), and severe (≤9) [12]. No prestroke cognitive decline: IQCODE <3.19 [13].

### 2.3. Imaging Protocol

Patients and the HC group were scanned once at admission and again at 1 year. MRI data were acquired using the same 3.0 T brain MRI (Achieva; Philips Healthcare, Best, Netherlands). The MRI protocol included 3D T1WI (TE/TR = 3.0/6.4 ms, acquisition matrix = 256 × 256, FOV = 256 × 256 mm^2^, slice thickness = 1 mm), 3D FLAIR (TE/TR/TI = 209 ms/4.8 s/1.65 s, acquisition matrix = 256 × 256, FOV = 256 × 256 mm^2^, slice thickness = 1 mm), DWI (TE/TR = 94/2,178 ms, acquisition matrix = 168 × 105, FOV = 220 × 204 mm^2^, slice thickness = 6.5 mm, *b* value = 0, and 1,000 s/mm^2^), and DKI (TE/TR = 70/4000 ms, acquisition matrix = 68 × 72, FOV = 200 × 224 mm^2^, slice thickness = 3 mm without gap). The DKI image was acquired with 3 *b*-values (*b* = 0, 1,000, and 2,000 s/mm^2^) along 15 diffusion gradient directions using a single-shot EPI sequence. The DKI acquisition covered the whole brain, with an acquisition time of 13 min 2 s.

### 2.4. MRI Processing

The infarct volume was constructed based on a semi-automatic segmentation on the DWI and FLAIR sequences using 3D Slicer 4.3.1 software (https://www.slicer.org). The Severity of WM hyperintensity (WMH) and MTA in patients and HC group were blindly evaluated by two radiologist in which disagreements of imaging analysis were resolved by consensus. WMH burdens were rated using the modified Fazekas scale [14] with periventricular hyperintensity (PVH) and deep WM hyperintensity (DWMH) each ranking from 0 to 3. MTA was rated using the Scheltens scale [15] ranging from 0 to 4, MTA score <1.5 was considered normal [16].

The DKI data were processed using the FMRIB's Software Library (FSL) (http://www.fmrib.ox.ac.uk/fsl) and Diffusional Kurtosis Estimator (DKE) (http://www.nitrc.org/projects/dke). First, eddy current distortions and intervolume subject motion were corrected using the eddy function of FSL. Second, DKE software was applied to calculate the FA, KFA, and MK. The TBSS method was performed on the previous parameters for statistical analysis. The FA maps of each subject were registered to the FMRIB58_FA template in the Montreal Neurological Institute (MNI) standard space using nonlinear transformations. The mean skeleton was created. The FA-threshold for the skeleton was >0.2 to include all major WM tracts and exclude superficial tracts and gray matter. Then, each participant's aligned images were projected onto the mean skeletons that were then used for voxel-wise statistical analysis. The disruption of WM tract integrity was defined by the decrease of KFA and/or MK.

### 2.5. Statistical Analysis

Statistical analysis was performed with SPSS version 22.0 software. Continuous variables were represented using either mean (standard deviation) or median (interquartile range, IQR), depending on whether they fit the normal distribution. Proportions were used to describe the categorical variables. The *t*-test or rank sum test was performed to compare continuous variables, while chi-square test was performed to compare categorical variables. A two-sided *P* < 0.05 was considered to be statistically significant.

PSCI was diagnosed according to the cognitive performance 1 year after stroke, and AIS group was subclassified with PSCI group and noncognitive impairment (NCI) group. We compared the differences in WM tract integrity between the AIS and HC groups, as well as between the PSCI and NCI groups at baseline and 1 year. We analyzed the WM tract integrity longitudinal alterations over the year in the AIS, PSCI, and NCI groups. Two-way repeated measures analysis of variance (ANOVA) were used to assess the difference in the evolution of WM tract integrity over the year between the PSCI and NCI groups.

Skeletonized DKI maps obtained from the TBSS approach were analyzed using the general linear model to test for group differences in major WM tracts. Randomized function in FSL was used for voxel-wise statistical tests. The models were corrected for gender, age, education, and NIHSS score. The threshold-free cluster enhancement option was used, and 5,000 permutations were generated. All statistical results were corrected for the family-wise error (FWE), and *P* < 0.05 was considered statistically significant.

## 3. Results

### 3.1. Demographics and Clinical Measurements

Eighty-five participants were enrolled after excluding poor imaging quality and incomplete clinical information. The median NIHSS score of AIS group at admission was 3 (IQR, 2–4). The median infarct volume at baseline was 0.87 ml (IQR, 0.67–2.22). Baseline characteristics and clinical data of participants are presented in Table 1. There were no significant differences in gender, age, education, body mass index, hyperlipidemia, diabetes, WMH, and PVH scores between the AIS and HC groups (*P* > 0.05). The DWMH score, prevalence of hypertension, and smoking in the AIS group were significantly higher than those in the HC group (*P* < 0.05). All participants had normal MTA scores, which were 0 (IQR, 0–0.5) at baseline and 0.5 (IQR, 0–1) at 1 year in the AIS group, and 0 (IQR, 0–0.5) at baseline and 0 (IQR, 0–0.5) at 1 year in the HC group.

### 3.2. Cognitive Function Assessment

**Figure 1:**
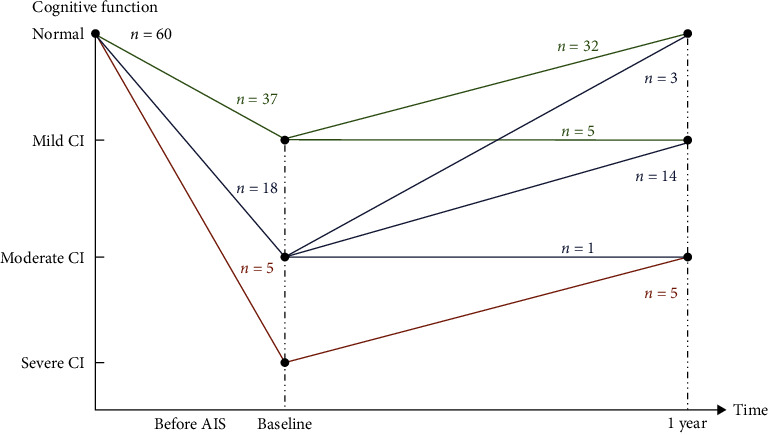
Cognitive changes in AIS group over the year poststroke.

All 25 participants in HC group had normal cognition at enrollment and 1 year. The MMSE and MoCA scores in AIS group were 21.83 ± 5.15 and 17.71 ± 4.92 (baseline), 25.88 ± 3.42, and 24.56 ± 5.11 (1 year), respectively, with significant differences (*P* < 0.01). There were 37 patients with mild CI, 18 cases with moderate CI, and 5 cases with severe CI in AIS group at admission assessment. One year after AIS, 25 patients were diagnosed as PSCI (PSCI group, including 19 patients with mild CI and 6 cases with moderate CI), 35 patients went from CI to normal cognition (NCI group). Compared with baseline, the severity of cognitive impairment improved in 54 patients and remained unchanged in 6 patients at 1 year. Cognitive changes in AIS group over the year poststroke are shown in Figure 1. Univariate analysis showed that there were significant differences between the PSCI and NCI groups in education, diabetes, and infarct location (*P* < 0.05) (Table 2). Multivariate logistic regression analysis indicated that strategic brain region infarct (thalamus, basal ganglia, frontotemporal lobes) was an independent risk factor for PSCI (odds ratio 0.17, 95% confidence interval 0.06–0.48, *P* < 0.05).

### 3.3. TBSS of the WM Tracts

#### 3.3.1. Comparison between the AIS and HC Groups

**Figure 2:**
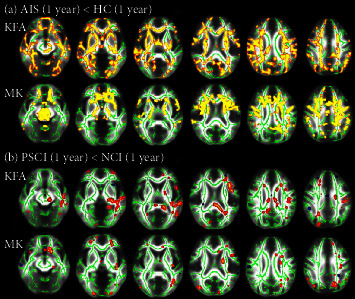
Groups differences in WM tract integrity. (a) Cluster in which less KFA and MK was detected in AIS group (1 year) compared with HC group (1 year) (*P* < 0.05, FWE-corrected). (b) Cluster in which less KFA and MK was detected in PSCI group compared with NCI group at 1 year (at 1 year) (*P* < 0.05, FWE-corrected). Regions showing significantly decreased parameter values (red and yellow) are overlaid on the mean skeleton (green).

There were no significant differences in KFA and MK of WM tracts between the AIS (at baseline) and HC groups (at baseline) (*P* > 0.05). The KFA and MK of multiple WM tracts in AIS group (1 year) were significantly lower than those in HC group (1 year) (*P* < 0.05), including superior longitudinal fasciculus (SLF), genu of corpus callosum (GCC), body of corpus callosum (BCC), splenium of corpus callosum (SCC), cingulum (CG), anterior thalamic radiation (ATR), posterior thalamic radiation (PTR), anterior limb of internal capsule (ALIC), posterior limb of internal capsule (PLIC), retrolenticular part of internal capsule (RLIC), sagittal stratum (SS), and fornix (FX). (Table 3, Figure 2(a)).

#### 3.3.2. Comparison between PSCI and NCI Groups

There were no significant differences in KFA and MK of WM tracts between the PSCI and NCI groups at baseline (*P* > 0.05). The KFA and MK of multiple WM tracts in PSCI group (1 year) were significantly lower than those in NCI group (1 year) (*P* < 0.05, Table 3, Figure 2(b)), including ATR, SLF, GCC, BCC, SCC, CG, PTR, ALIC, RLIC, SS, and superior fronto-occipital fasciculus (SOF).

#### 3.3.3. Longitudinal Analysis in Each Group

**Figure 3:**
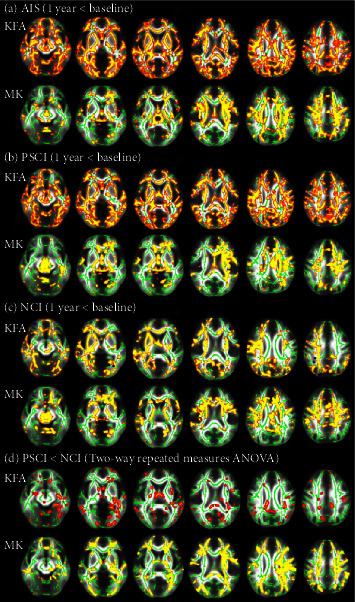
Longitudinal alterations in WM tract integrity. Cluster in which less KFA and MK was detected at 1 year compared with baseline in the AIS (a), PSCI (b), and NCI groups (c). Two-way repeated measures ANOVA showed that PSCI group had higher KFA and MK decrease over the year of follow-up than NCI group (d) (*P* < 0.05, FWE-corrected). Regions showing significantly decreased parameter values (red and yellow) are overlaid on the mean skeleton (green).

Longitudinal analysis showed that the KFA and MK of multiple WM tracts recorded at 1 year were significantly lower than those recorded at baseline in the AIS, PSCI, and NCI groups (*P* < 0.05, Table 4, Figure 3(a)–3(c)). Two-way repeated measures ANOVA showed that PSCI group had higher KFA and MK decrease in multiple WM tracts over the year of follow-up than NCI group (*P* < 0.05, Table 4, Figure 3(d)). There were no significant differences in KFA and MK of WM tracts recorded at baseline and 1 year in the HC group (*P* > 0.05).

## 4. Discussion

To understand the impact of AIS on the risk of PSCI, prestroke cognitive status must be taken into consideration. As Alzheimer's disease pathology is prevalent among older people, the selected population in this study were relatively young and all cases were assessed by IQCODE to exclude patients with CI before stroke. MTA is the most prevalent early imaging marker of Alzheimer's disease [17]. The MTA scores of AIS group at baseline and 1 year were normal, which also indicated that the patients did not coexist with neurodegenerative diseases before stroke. The DWMH score, prevalence of hypertension and smoking in AIS group were significantly higher than those in HC group, indicating that patients had higher vascular risk factors.

One year poststroke, of the 60 original patients, 25 were diagnosed as PSCI and 35 recovered to normal cognition, indicating a 41.7% PSCI incidence. We found no significant difference in infarct volume between the PSCI and NCI groups, which may be due to the fact that the majority of patients had relatively small acute lesions rather than large infarcts. Infarct location is an important determinant for PSCI. Strategic infarcts often occur in the thalamus, hippocampus, basal ganglia, angular gyrus, medial frontal lobe, inferomedial portion of the temporal lobe. Strategic infarction dementia may be caused by damage to the components of Papez or Yakovlev circuits [18, 19]. TBSS analysis showed that the KFA and MK of multiple WM tracts in PSCI group were significantly lower than those in NCI group at 1 year. The ATR consists of connecting fibers that link the anterior thalamic nucleus with anterior cingulate gyrus and the mediodorsal thalamic nucleus with frontal cortex. It participates in the prefrontal subcortical circuit, which is considered to be correlated with processing speed and executive function [20]. The SLF is composed of long bidirectional frontal–parietal–temporal–occipital projections among the prefrontal, temporal, and occipital cortices via the posterior parietal cortex, and it is critical for visuospatial cognitive function and working memory performance [21]. The corpus callosum passes signals to various regions of the contralateral hemisphere cortex; corpus callosum lesion is associated with reduced cognitive performance in the domains of information processing speed, executive functioning, memory, and visuospatial processing [22]. The disruption of the CG can lead to the disruption of communication from the cingulate gyrus and hippocampus to the cerebral cortex, subsequently impairing episodic memory and other cognitive domains. The commissural fibers between cortical areas of the same hemisphere, association fibers between each hemisphere, and projection fibers between cortex and subcortical regions participate in neuronal circuits related to cognitive function, and their damages correlate to PSCI [23].

The cognitive performance of patients had improved significantly over the year. However, longitudinal analysis showed that WM tract integrity of AIS group deteriorated at 1 year compared with baseline, and similar deterioration could also be seen in PSCI group. It seems as if the alterations of WM microstructure at baseline and 1 year had been inconsistent with the corresponding cognitive performance of patients.

In fact, PSCI is triggered by vascular events and is partially recoverable. Although the early cognitive score poststroke tends to be low, improvement is often seen after the acute stage [1, 2] due to revascularization and brain plasticity [24]. Therefore, the final diagnosis of PSCI should be delayed to at least 6 months after the event [25], which leaves sufficient time for the recovery of brain function and helps to find late-onset PSCI. In this study, 54/60 patients improved their cognition 1 year poststroke, which may be due to their younger age and vascular event as their main pathogeny. It has been reported that younger age and less neurodegenerative factors involved were associated with favorable cognitive outcomes [3].

Pathologically, in the hyperacute stage of AIS, local ischemia and hypoxia of tissue cause cytotoxic edema and a decrease of extracellular fluid without a significant change in structural integrity, which may result in increased directionality of diffusion along the axon; FA will increase slightly or not decrease [26]. As time goes on, loss of myelin and axonal disruption will occur, leading to the damage of nerve fiber integrity, the point at which FA and MK begin to decrease [4, 5]. Diffusion MRI studies have shown the disruption of WM integrity at 14 days [7], 1 month [5], and 3 months [8] poststroke and its impact on cognition. TBSS analysis showed that no significant differences of WM tract integrity were found between the AIS (at baseline) and HC groups. Also, there were no significant differences of WM tract integrity between the PSCI and NCI groups at baseline. Our results support Cha's study that the disruption of WM tract integrity in the early acute stage may not be significant [26].

Although age-related WM degeneration in aging is a well-known process, we find no deterioration in WM microstructure and cognitive performance over the year of follow-up in HC group. Therefore, we believe that for a relatively young population, one year may be insufficient to cause WM microstructural alteration. We cannot exclude the possibility that the deterioration of WM integrity in patients was due to stroke. In vivo study in animal experiments has demonstrated that anterograde Wallerian and/or retrograde axonal degeneration following AIS can injure descending and ascending fibers [27], resulting in disruption of WM tract integrity bilaterally remote from the infarct with such damage possibly developing gradually over time [8]. Also, we found that PSCI group had a faster WM degeneration than NCI group, which is consistent with Sagnier's data [4].

In this study, 35/60 patients with baseline impaired cognitive functions returned to normal cognition 1 year later. Compared with baseline, it was found that these patients still displayed disruption of WM tract integrity 1 year poststroke, although such damage was insufficient to cause CI symptoms. This suggests that cognitive vulnerability at baseline is associated with an increased radiological frailty condition 1 year poststroke [4]. The effect of this WM microstructural alteration on long-term outcomes beyond 1 year should be considered. In a follow-up study on the transitions of the cognitive status of 109 patients from 1 to 7 years poststroke [3], 33 patients had cognitive deterioration, which 19 patients went from normal cognition to mild CI, 12 from mild CI to dementia, and 2 from normal cognition to dementia. Therefore, we should continue to pay attention to subsequent changes in these patients, withdraw from the apparition of WM macrostructural abnormalities, and prevent cerebrovascular events from triggering or accelerating underlying neurodegenerative processes [2]. It has been reported that the modification of cardiovascular risk factors, psychosocial factors, and healthy behaviors can slow down the negative impact on WM microstructure [24, 28].

This study had some limitations: First, limited by patient inclusion criteria, the sample size of this study is limited, which may lead to sampling bias and affect relevant conclusions. Second, this study defines CI according to MMSE and MoCA scores. Although they allowed a rapid screening of cognitive impairment, some patients with cognitive decline might not be identified. More exhaustive neuropsychological batteries should be considered in further studies.

## 5. Conclusions

The disruption of WM tract integrity in the early acute stage of AIS may not be significant. Although the cognitive performance of patients with baseline impaired cognitive functions has improved or returned to normal at 1 year poststroke, there is still WM microstructural damage, which may have a long-term impact on cognition. Further researches are needed to investigate WM integrity evolution beyond 1 year and its relation to cognitive outcome.

## Figures and Tables

**Table 1:** Clinical characteristics of the AIS and HC groups.

Variables	HC group (*n* = 25)	AIS group (baseline) (*n* = 60)	*P*
Demographic characteristics			
Gender (male), *n*	16	42	0.588
Age (year), mean (SD)	56.16 (6.96)	57.70 (7.33)	0.337
Education (year), mean (SD)	12.56 (3.22)	11.23 (3.90)	0.080
Vascular risk factors			
Body mass index, mean (SD)	24.79 (2.83)	25.60 (2.71)	0.183
Hypertension, *n*	13	49	0.008
Hyperlipemia, *n*	9	34	0.082
Diabetes, *n*	5	16	0.516
History of smoking, *n*	10	43	0.006
Cognitive scores			
MMSE score, mean (SD)	28.80 (1.11)	21.83 (5.15)	<0.001
MoCA score, mean (SD)	27.36 (1.38)	17.71 (4.92)	<0.001
Radiological data			
WMH scale, median (IQR)	2 (2–2.5)	2 (2–3)	0.054
PVH scale, median (IQR)	1 (1–1.5)	1 (1–2)	0.086
DWMH scale, median (IQR)	1 (1)	1 (1)	0.025
MTA score, median (IQR)	0 (0–0.5)	0 (0–0.5)	–

Abbreviations: MMSE, mini-mental state examination; MoCA, Montreal Cognitive Assessment; WMH, white matter hyperintensity; PVH, periventricular hyperintensity; DWMH, deep white matter hyperintensity; MTA, medial temporal lobe atrophy; SD, standard deviation; IQR, interquartile range.

**Table 2:** Univariate analysis of clinical characteristics between the PSCI and NCI groups.

Variables	PSCI group (*n* = 25)	NCI group (*n* = 35)	*P*
Gender (male), *n*	17	25	0.775
Age (year), mean (SD)	57.32 (8.16)	57.97 (6.78)	0.699
Education (year), mean (SD)	9.80 (3.56)	11.86 (3.72)	0.011
Body mass index, mean (SD)	25.96 (2.93)	25.35 (2.56)	0.322
Hypertension, *n*	19	30	0.067
Hyperlipemia, *n*	14	20	0.930
Diabetes, *n*	10	6	0.048
History of smoking, *n*	17	26	0.594
WMH scale, median (IQR)	3 (2–3)	3 (2–3)	0.797
PVH scale, median (IQR)	2 (1–2)	2 (1–2)	0.960
DWMH scale, median (IQR)	1 (1)	1 (1)	0.548
Strategic infarct location (yes/no), *n*	20/5	13/22	0.001
Infarct volume (ml), median (IQR)	0.87 (0.66–2.80)	0.91 (0.68–1.77)	0.865

Abbreviations: WMH, white matter hyperintensity; PVH, periventricular hyperintensity; DWMH, deep white matter hyperintensity; SD, standard deviation; IQR, interquartile range.

**Table 3:** WM tracts showed significant differences in KFA and MK between groups in TBSS analysis.

Parameters	Index	Voxels	Peak MNI coordinates			*P*	Major tracts^a^
			*X*	*Y*	*Z*		
AIS (1 year) < HC (1 year)							
KFA	1	65,037	2	25	12	0.002	SLF, GCC, BCC, SCC, CG, ATR, PTR, ALIC, PLIC, RLIC, SS, FX
MK	1	26,926	33	−7	21	0.005	SLF, GCC, BCC, SCC, CG, ATR, ALIC, PLIC
PSCI (1 year) < NCI (1 year)							
KFA	1	962	0	−36	15	0.008	SCC, PTR, RLIC, CG, SS
	2	418	−41	−23	30	0.002	SLF
	3	243	−15	3	33	0.012	BCC
	4	66	10	−45	27	0.022	CG
	5	23	−21	32	15	0.034	ATR
MK	1	148	−21	−6	19	0.020	SOF, ATR, ALIC
	2	51	−35	−24	−2	0.015	RLIC
	3	50	−31	−66	1	0.015	PTR
	4	32	−10	20	16	0.035	GCC
	5	26	−30	−23	40	0.025	SLF

Abbreviations: MNI, Montreal Neurological Institute; SLF, superior longitudinal fasciculus; GCC, genu of corpus callosum; BCC, body of corpus callosum; SCC, splenium of corpus callosum; CG, cingulum; ATR, anterior thalamic radiation; PTR, posterior thalamic radiation; ALIC, anterior limb of internal capsule; PLIC, posterior limb of internal capsule; RLIC, retrolenticular part of internal capsule; SS, sagittal stratum; FX, fornix; SOF, superior fronto-occipital fasciculus. *P*: threshold-free cluster enhancement, FWE-corrected. ^a^JHU white-matter tractography atlas.

**Table 4:** WM tracts with significantly less KFA and MK at 1 year compared with baseline in longitudinal analysis.

Parameters	Index	Voxels	Peak MNI coordinates			*P*	Major tracts^a^
			*X*	*Y*	*Z*		
AIS (1 year < baseline)							
KFA	1	104,046	−8	−22	27	0.002	SLF, GCC, BCC, SCC, CG, ATR, PTR, ALIC, PLIC, RLIC, SS, FX, UF, CST
MK	1	19,675	−12	−29	25	0.005	SLF, GCC, BCC, SCC, SOF, ATR, ALIC, RLIC, CST
	2	10,902	34	−18	25	0.005	SLF, CG, PLIC
	3	2,557	1	−17	18	0.005	FX
	4	258	−40	−40	−9	0.010	SS
	5	250	−32	−65	3	0.015	PTR
PSCI (1 year < baseline)							
KFA	1	96,165	−5	−13	27	0.002	SLF, GCC, BCC, SCC, CG, ATR, PTR, ALIC, PLIC, RLIC, SS, FX, UF, CST
MK	1	17,936	1	9	21	0.005	SLF, GCC, BCC, SCC, CG, ATR, PTR, ALIC, PLIC, RLIC, SS, SOF, FX, CST
NCI (1 year < baseline)							
KFA	1	46,526	9	22	−5	0.002	SLF, GCC, BCC, SCC, CG, ATR, PTR, ALIC, PLIC, RLIC, SS, FX, CST
MK	1	32,281	1	26	−1	0.005	SLF, GCC, BCC, SCC, CG, ATR, ALIC, PLIC, RLIC, SOF, CST
PSCI < NCI (two-way repeated measures ANOVA)							
KFA	1	645	12	−36	25	0.005	BCC, SCC, PTR
	2	540	−20	−35	−11	0.005	CG, SS, PLIC, RLIC
	3	216	−13	29	−11	0.005	ATR
	4	191	−35	−29	31	0.005	SLF
	5	172	18	15	2	0.020	ALIC, FX
	6	97	18	25	22	0.010	GCC
MK	1	14,384	−31	3	21	0.005	SLF, GCC, BCC, SCC, ATR, PTR, SOF, ALIC, PLIC, RLIC, CG, SS

Abbreviations: MNI, Montreal Neurological Institute; SLF, superior longitudinal fasciculus; GCC, genu of corpus callosum; BCC, body of corpus callosum; SCC, splenium of corpus callosum; CG, cingulum; ATR, anterior thalamic radiation; PTR, posterior thalamic radiation; ALIC, anterior limb of internal capsule; PLIC, posterior limb of internal capsule; RLIC, retrolenticular part of internal capsule; SS, sagittal stratum; FX, fornix; UF, uncinate fasciculus; CST, corticospinal tract; SOF, superior fronto-occipital fasciculus. *P*: threshold-free cluster enhancement, FWE-corrected. ^a^JHU white-matter tractography atlas.

## Data Availability

The original contributions presented in the study are included in the article material; further inquiries can be directed to the corresponding authors.
